# Monte Carlo optimum management of ^241^Am/Be disused sealed radioactive sources

**DOI:** 10.1038/s41598-022-05221-y

**Published:** 2022-01-21

**Authors:** Cebastien Joel Guembou Shouop, Serge Mbida Mbembe, Cedric Tayou Kamkumo, Jean Felix Beyala Ateba, Maurice Ndontchueng Moyo, Eric Jilbert Nguelem Mekongtso, Augustin Simo, David Strivay

**Affiliations:** 1 UFD Mathématiques, Informatique Appliquée et Physique Fondamentale, Université de Douala, P.O. Box 24157, Douala, Cameroun; 2 National Radiation Protection Agency of Cameroon, Box 33732, Yaounde, Cameroon; 3 Atomic and Nuclear Spectroscopy, Archeometry, University of Liège, Bat. B15 Sart Tilman, 4000 Liege 1, Belgium

**Keywords:** Applied physics, Nuclear physics, Particle physics

## Abstract

The optimum encapsulation of ^241^Am/Be disused sealed radioactive sources (DSRS) based on PHITS Monte Carlo simulations for their long-term storage in Cameroon was performed. The country capacity for the management of disused neutron sources was also evaluated and showed that a Am1 P60 capsule is sufficient for the total available inventoried ^241^Am/Be DSRSs. The effective dose rate was computed in the enclosures of the DSRS container, which will be temporarily stored in the centralized radioactive waste facility. The obtained results were in agreement with the ALARA principle for the exposure rate optimization and the obtained exposure dose rates were found to be 1.830 μSv/h (horizontal calculation) and 0.137 μSv/h (vertical computation) which values are lower than the 2.5 μSv/h acceptable limit for the public area. The dose profile for ^241^Am/Be source obtained, the neutron flux, and gamma generated from neutron absorption showed agreement with the research hypothesis. The Monte Carlo assessment achieved in the present research will be useful for dismantling and preparing the waste package for long-term storage.

## Introduction

Recently, the use of sealed radioactive sources (SRSs) in industrial applications such as petroleum exploration (oil and gas industries), well logging, and non-destructive tests has been increased worldwide. At their end-life-cycle, the SRSs can be recycled or reused for other purposes. If a country does not have the capability to do so, they become disused sealed radioactive sources (DSRSs) and need to be safely managed. In developing countries like Cameroon, the inventory of radioactive sources available at the National Regulatory Authority revealed an increase number of disused radioactive sources over the years. The management of DSRSs is acknowledged as a real challenge^1–9^. One of the management options is to send back to the manufacturers DSRS that cannot be used furthermore, but at time, this option is not possible and the country where it was being used should develop its own management capability. Safe management of these disused low activity radioactive sources has been a great challenge especially for neutron sources where shielding requires appropriate materials. As a result, Regulatory Authorities of developing countries have established and maintained in collaboration with international organizations and nuclear power country’s regulatory authorities as the Department of Energy of the United States of America (or USNRC) and the International Atomic Energy Agency, a temporary radioactive waste management facility.

When a radioactive source becomes disused, it is the most vulnerable part of its life cycle as the control procedures change because of being not profitable to the end-users. But it is a sensitive stage because of accident and/or incidents that can result if the disused source is being neglected^10–15^. Although an improvement has been made in safe management of disused sealed sources in developing countries, the issue regarding the space security management has also been another challenge. Nevertheless, research works have been carried on developing advanced technology for dismantling and disposal of several sources into a special capsule and container^16,17^.

Through technical cooperation with its member’s states, the IAEA has developed and established projects on management of disused sealed radioactive sources entitled “Sustaining Cradle-to-Grave Control of Radioactive Sources—Phase II”^18–20^. The project aims to assist developing countries to acquire appropriate knowledge on the management of DSRSs. Though most countries have laid down regulatory framework on control of sealed sources, the management of DSRSs remains an up-to-date scientific topic^21–26^. There are still several uncertainties on DSRS containers used for radioactive waste disposal since DSRSs emit neutron and gamma radiations, known to cause high level risk if no appropriate protective measures are undertaken. Monte Carlo simulations are used to optimize the DSRS disposal containers with a view to reduce the high radiological risk to the population and to the radioactive waste management staff.

Since neutron emitters as ^241^Am/Be have a great variation of stochastic characteristics, their domestic long-term storage should be considered for optimizing the dismantling and disposal method. Such complex operation should not be mistakenly implemented and one of the most powerful tools that can be used to evaluate the waste package container radiological safety is the Monte Carlo computational method^8,9,27–32^. In the present study, the optimum encapsulation of ^241^Am/Be disused sources based on Monte Carlo simulations for the disposal of DSRSs has been suggested. The Monte Carlo project is implemented based on the Particle and Heavy Ion Transport code System (PHITS) before the on-site dismantling and long-term storage operations are undertaken^31,33–38^.

## Monte Carlo simulation

Monte Carlo methods also named MC techniques are usually acknowledged as a group of computational algorithms based on the principle of repetition of random sampling to estimate unknown parameters and that use random number generators to solve problems that are stochastics in nature. MC methods are effective in modeling complex situations and problems with a high degree of freedom that cannot be analytically solved, by performing random numbers and random experiments associated with a specific probability density function^27,32,39^. The application of MC to neutron transport is one of the most appropriate means to evaluate neutron interaction in a medium since the neutron interaction with matter (or a medium) is a cross-section-dependent function. This radiation (Neutron) interaction with matter usually involved parameters such as the microscopic cross-section (σ), which describes the interaction of neutron with a light particle and is expressed in the unit of barn or cm^−2^. Then, when neutrons interact with heavy materials or compounds such as a wall, concrete, or macroscopic material, the involved parameter is the macroscopic cross-section (∑). Therefore, the total interaction neutron cross-section in a medium in MC simulations is considered as the summation of all cross-sections including the absorption, scattering, fission, capture, etc.^3^. The following equations, therefore, describe the macroscopic cross-section of neutron interaction in our geometry during the simulation:

1 $$\Sigma = N\,\,\sigma$$

2 $$\Sigma_{Total} = \Sigma_{Scattering} + \Sigma_{Absorption} + \Sigma_{Capture} + \Sigma_{Fission} + ...$$

3 $$N = \frac{\rho }{A}\,\,N_{A}$$

In the previous equations, N describes the atomic density of the target material, ρ refers to the target material density, and *N*_*A*_ is the Avogadro’s number. From the equation, one can recall that materials with large total cross-sections are good neutron moderators. But if the source emits neutrons with energy higher than 2 MeV, the most important parameter is the removal cross-section that should be considered physically in the simulation. This is mainly because the fast neutrons need to be thermalized before being efficiently absorbed in a medium.

As the main concern in dealing with DSRSs is the neutron shielding, three steps should be considered as the average neutron energy is 4.5 MeV: (i) slow the neutrons, (ii) absorb the neutrons, and (iii) absorb the gamma rays generated from neutron interaction. To slow neutrons emitted from ^241^Am/Be source to thermal energies, light or hydrogenous materials (water that can evaporate or leak, flammable paraffin, or plastic) are usually used. To absorb neutrons, hydrogenous materials are also effective but gamma-ray emission is difficult to shield, and it is appropriate to use boron or borate material. In other to effectively evaluate the shielding property of a waste capsule or container, another important parameter to be considered for neutron shielding is the mean free path denotes by λ. This parameter is described as the average distance a neutron travels between two collisions in the target material. There is therefore a way to express the collisional probability at a distance dx taken by a neutron in a medium^3^:

4 $$p(x)\,\,dx = \sum\limits_{t} {dx.e^{{ - \Sigma_{t} }} } .\,x = \sum\limits_{t} {e^{{ - \Sigma_{t} }} } .\,x.\,\,dx$$

5 $$\lambda = \int\limits_{0}^{\infty } {p\,(x).x\,.dx} = \frac{1}{{\Sigma_{t} }}$$

Neutron transmission, that is the ratio between the intensity of incoming neutrons and that of neutrons passing throughout the medium, determines the properties of the material used and its ability to slow down and absorb radiation. For the Monte Carlo modeling in this study, the Particle and Heavy Ion Transport code System (PHITS) version 3.22 was used. It is a general-purpose Monte Carlo code developed using Fortran programming language. The code was used to validate the model built in the present study and to verify the effectiveness of material used in slowing down and absorbing neutron emitted from ^241^Am/Be DSRSs^31,33,37^. That ability is related to the distance λ and the removal cross-section. PHITS’s use for waste package design, radiation shielding, and radiation protection requires an appropriate well-defined method. In this regard, the calculation validation is based on the relative standard deviation calculation when the input code is well set. The following equation was implemented by the PHITS research team to evaluate the uncertainties on the computed values:

6 $$\sigma = \sqrt {\frac{{\sum\limits_{i = 1}^{N} {\left( {{\raise0.7ex\hbox{${x_{i} w_{i} }$} \!\mathord{\left/ {\vphantom {{x_{i} w_{i} } {\overline{w}}}}\right.\kern-\nulldelimiterspace} \!\lower0.7ex\hbox{${\overline{w}}$}}} \right)^{2} } - N\overline{X}^{2} }}{N(N - 1)}}$$

The input file built in the present research was compiled and executed using the Radioactive Decay Process, a data library from DECDC2 (Nuclear Decay Data for Dosimetry Calculation, version 2), a revised data package from ICRP Publication 38. The library allows the end-user to use a precompiled system for 1034 nuclides for dose calculation in medical, environmental and occupational exposures. This package is a library built from the assembled set of Evaluated Nuclear Structure Data File (ENSDF)^9,34,40–42^.

## Material and methods

Nuclear waste management is one of the most challenging issues in nuclear engineering and advanced countries deal with spent nuclear fuel while states with no nuclear power plants have to manage DSRSs. In either case, disused neutron emitters are involved. Since the present study aims to provide an optimized Monte Carlo-based DSRSs management, the description of the neutron source, the geometry, and a brief description of the input code is given below.

### ^241^Am/Be neutron source simulated

The DSRS consisted of isotropic (α, n) ^241^Am/Be neutron source. The α-emitter is the Am-241, the target is Be-9, the Q-value of the reaction is 5.71 MeV and the neutron yield per α is ~ 7.0E(-5) for the following reaction^43^:

7 $$\begin{gathered} {}_{{92}}^{{238}} U\,\, + \,{}_{2}^{4} He\,\,\,\, \to \,\,{}_{{94}}^{{241}} Pu\, + \,\,{}_{0}^{1} n\, \hfill \\ {}_{{94}}^{{241}} Pu\,\,\,\,\xrightarrow{{\beta ^{ - } }}\,{}_{{95}}^{{241}} Am\, \hfill \\ \end{gathered}$$

where the neutron produced per unit energy interval in Be (α, n) sources can be estimated using the following equation:

8 $$n(E)\,\, = \,\,2\,\pi \,\sigma (E)\,\,\sin \theta \,\,\frac{d\theta }{{dE}}\,\,\frac{{\Delta E_{\alpha } }}{{\varepsilon (E_{\alpha } )}}$$

where *σ(E)* refers to the differential cross-section for neutron production in the center of mass, *ε* is the stopping cross-section for Be, and *E*_*α*_ the alpha particle energy.

**Figure 1:**
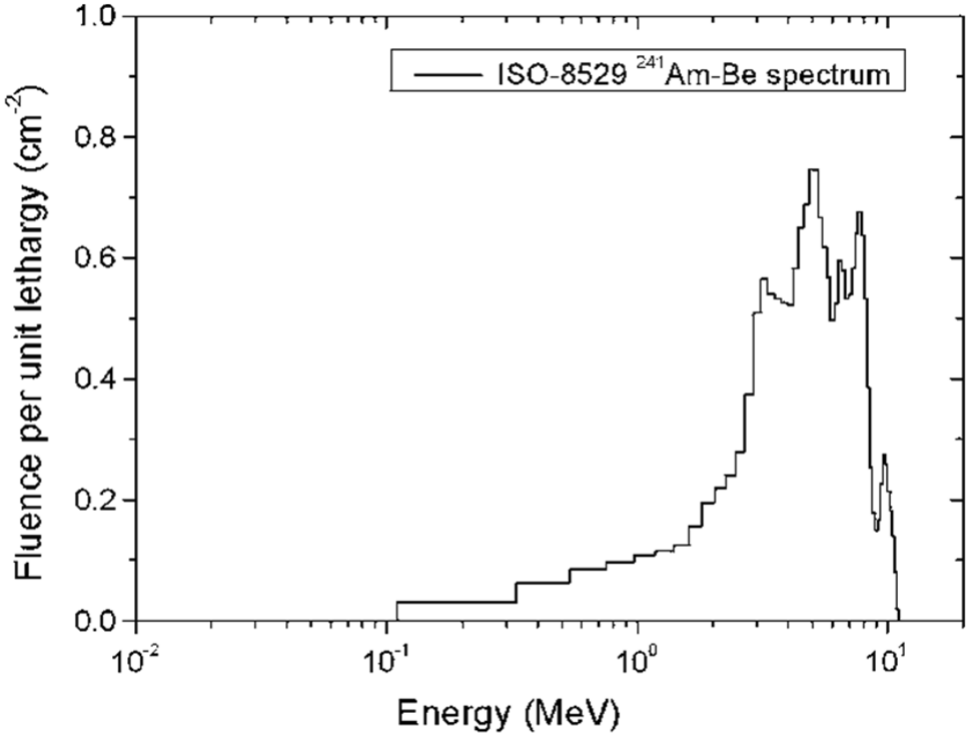
^241^Am/Be neutron spectrum from ISO 8529 used for Monte Carlo Simulation. The PHITS input code uses this spectrum for the neutron emission from DSRSs under investigation.

The ^241^Am/Be DSRS used in the present work is made of 8 disused sealed neutron sources that will be dismantled and safely disposed of during the expert mission. The characteristics of the eight composite sources are presented in Table 1 along with their activities and the spectrum of the source implemented is given in Fig. 1. The source definition was done in PHITS input code using ispfs parameter for spontaneous fission as ^241^Am is one of the 18 fission nuclei defined in PHITS code. A volume source (cylindrical) was defined inside the P60 capsule prepared to contain the dismantled DSRSs. The energy spectrum of the ^241^Am/Be source used is shown in Fig. 1. Regarding the neutron emission rate, Strain^44^ measured an emission rate of 5.7 × 10^6^ neutrons/second in 1962. In 2019, M. Tohamy et al. in their study, expected neutron yield of (2.2 ± 0.2) × 10^6^ s^-1^ Ci^-1^^45–49^. These details were implemented for the source definition in the present study using neutrons with a mean energy of 4.5 MeV in association with 4.438 MeV γ-rays according to the reaction ^241^Am (n, γ) ^12^C.

**Table 1:** List of disused sealed radioactive sources of ^241^Am/Be considered for one P60 capsule and projected for the dismantling operation and long-term storage activity. The estimated activities were evaluated using the datasheet from the manufacturers, that is the radioactive decay law.

Manufacturer	Number of sources	Nominal activity per source (mCi)	Date of activity	Estimated activity per source on 08/16/2021 (mCi)
TROXLER	02	40.0 ± 0.8	16-08-1982	37.57 ± 0.74
HUMBOLT	04	40.0 ± 0.8	13-09-1989	38.00 ± 0.75
SEDITECH	02	50.0 ± 1.0	15-06-1987	47.33 ± 0.95
**Total**	**08**			**321.82**

### Geometry of the neutron DSRS package

**Figure 2:**
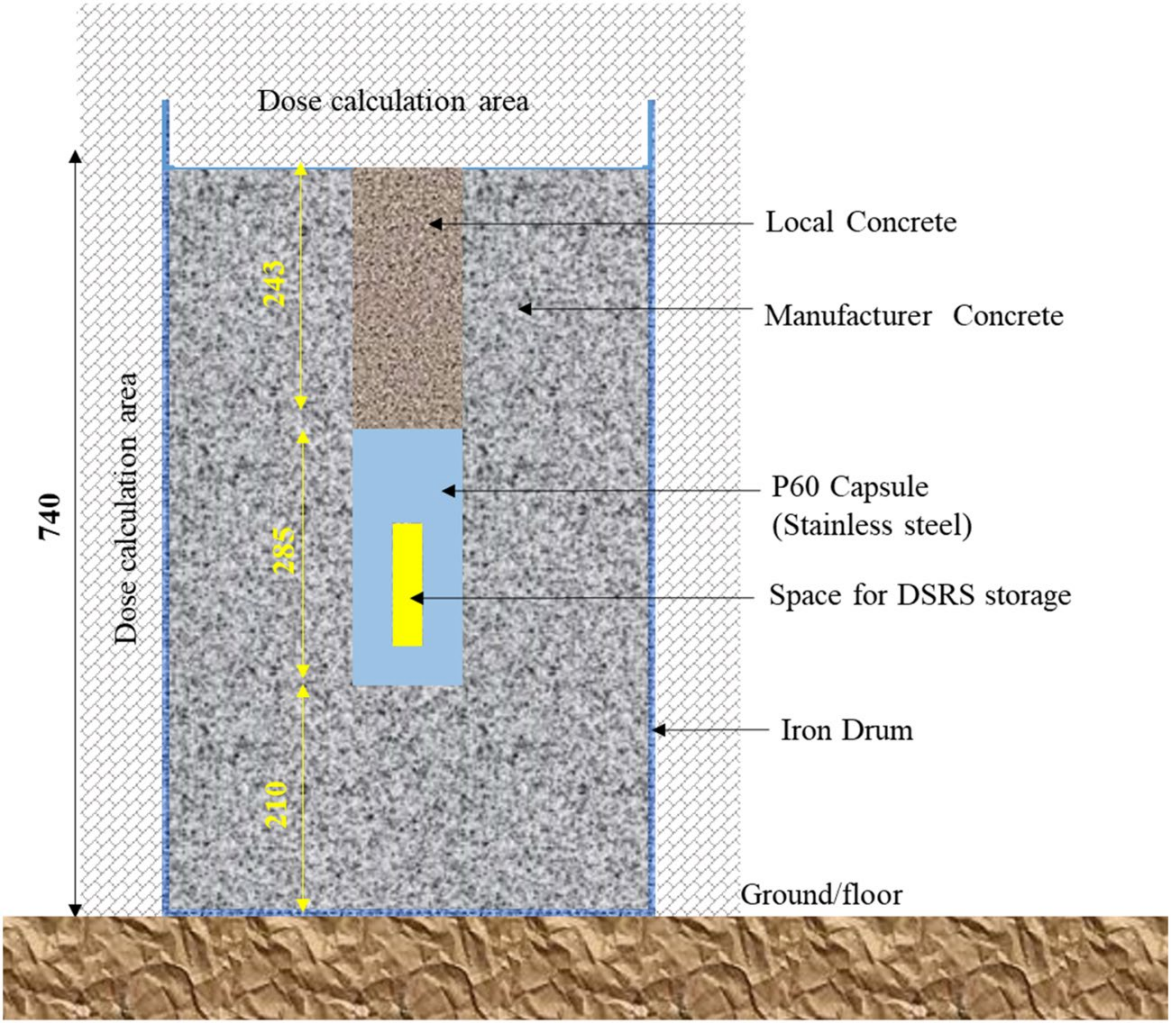
Geometry of the simulation designed for DSRSs waste management optimization. The effective dose calculation area is set on the r-axis (refer to as the x-axis or horizontal axis) and z-axis (refer to as the vertical axis in the remaining part of the manuscript).

The geometry of the system used to contain DSRSs after dismantling for their disposal is shown in Fig. 2. The DSRSs were dismantled and ^241^Am/Be sources were stored in the Am1 P60 Capsule, then the P60 capsule was encased into a well-prepared borate concrete drum as presented in Fig. 2. The entrance was sealed then cover with locally made concrete that is less effective than the industrial made concrete. The Am1 P60 capsule is made of stainless steel with an outer wall thickness of 10 mm, 68 mm diameter, and with a length or height of 285 mm, sealed with a tight conical plug, pressed into the conical neck using a threaded cap.

**Figure 3:**
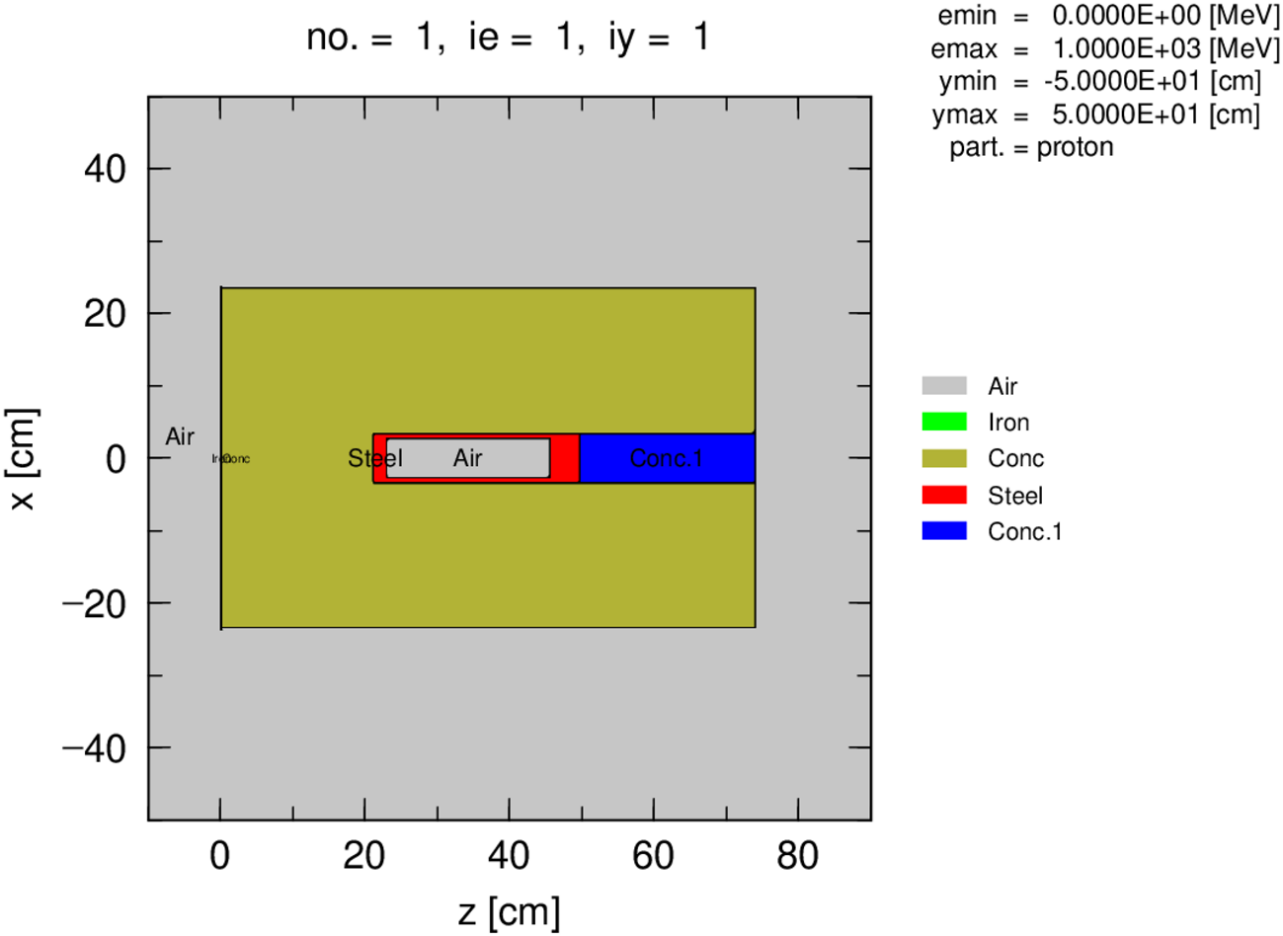
Output Geometry of the simulation designed for DSRSs waste management optimization generated by PHITS. Different components of the geometry with the materials used and their dimensions.

The output geometry computed using PHITS code is shown in Fig. 3 where each material used for the geometry design is represented by a different color in the legend. The tallies for dose calculation and decision-making process were set one meter away and at the contact of the capsule in two directions: the vertical that is the z-axis and the horizontal, which is r-axis (by extension x-axis due to the isotropic emission of the source). The importance was set throughout the geometry depth as the variance reduction method allows fast calculation and accurate results achievement in a Monte Carlo calculation.

### The PHITS input code

The PHITS Monte Carlo input code for the present study includes the following sections: Title, Parameters, Source, Material, MatNameColor, Surface, Cell, T-3Dshow, Importance, three T-Track sections, Multiplier, and End^34,50–52^. The T-Track sections define different physical values that should be extracted during the simulation in other to discuss the obtained data. The range of interest of space and energy as well as the unit of each physical parameter was set. The results presented in the following section were obtained from a validated input code. In the PHITS input code, the variance reduction is implemented using the weight windows tally. The variance reduction can therefore be used more effectively by biasing the [weight window] parameter for a specific region with [ww bias]^53^.

## Results and discussions

**Figure 4:**
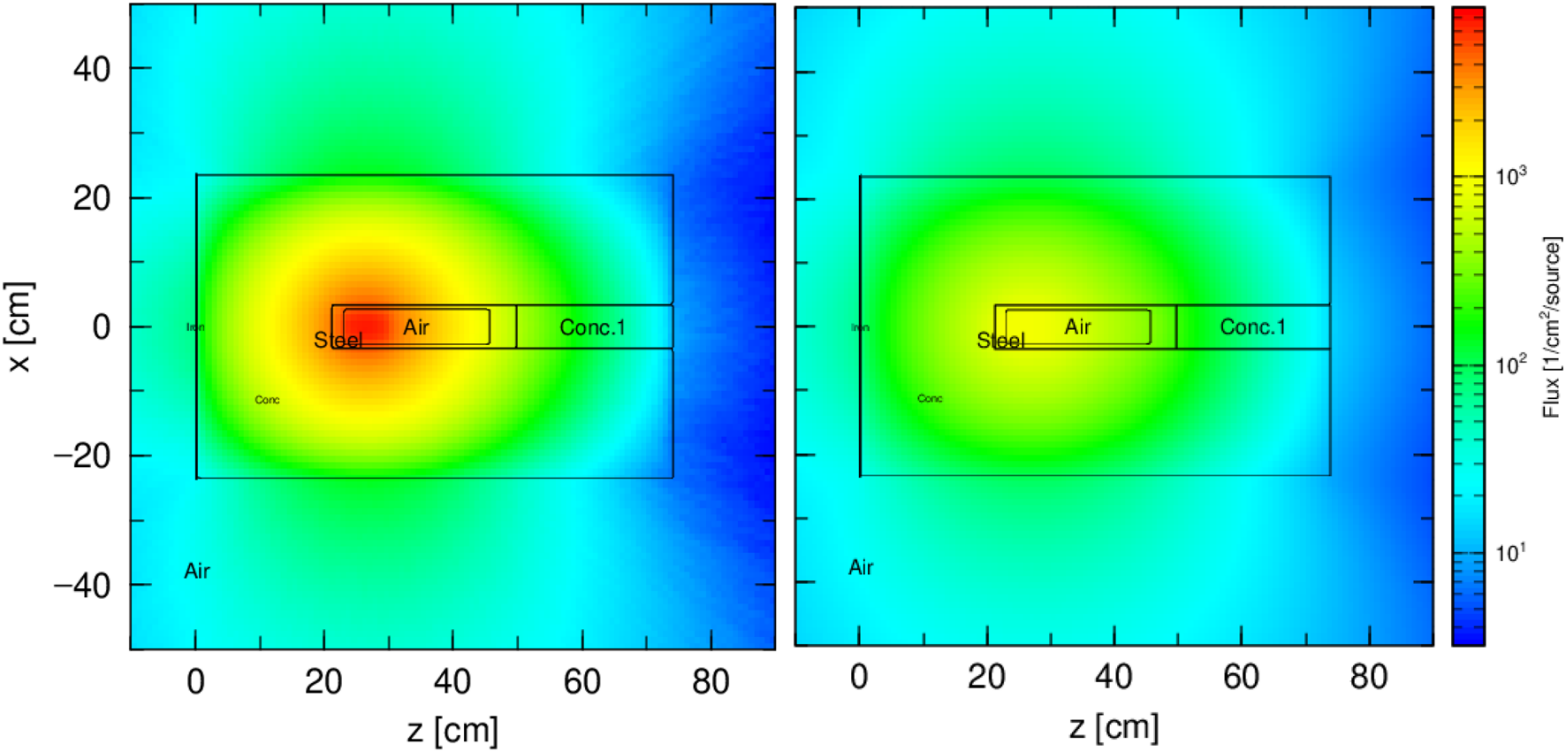
Neutron flux (left) and generated photon flux (right) obtained in the computed geometry from the DSRSs waste made of the compilation of ^241^Am/Be sources in a P-60 capsule and stored in a barite concrete-filled drum. Monte Carlo view for a run of 5 000 000 initial experiences.

Normalized neutron and photon fluxes are presented in Fig. 4. The neutron flux is highest at the ^241^Am/Be source position which is confined at the Am1 P60 capsule center. The remaining space in the P60 capsule is filled with air in the simulated model, as presented in the previous section. As can be seen in Fig. 4, the neutron flux is symmetric to the z-axis and decreases gradually towards the outside of the shielding geometry, as neutrons are adsorbed by the concrete-filled drum and P60 capsule. Compared to the neutron flux, the photon flux is about one order magnitude lower at the center of the geometry. But when neutrons undergo total absorption at the exit cross-section of the shielding geometry, the photon flux increase to be higher than the neutron flux. This is observed by comparing the top (z-axis) of the geometry where the dark blue color represents the lowest flux for both neutron (dark blue) and photon (light dark blue). Such effect is due to the fact that photon shielding requires high-Z materials such as lead, whereas neutrons are effectively shielded by hydrogen-rich materials such as water, paraffin, borate concrete. For this reason, neutron shielding always involved gamma shielding material set at a position where the neutron absorption reaches its paroxysm to effectively shield the gamma generated from neutron absorption. This can be observed at the position (x; z) = ([− 50 + 50]; [80 90]) in the unit of cm. In addition, while appropriate materials for gamma shielding are not effective for neutron shielding, neutron shielding materials are ineffective for gamma radiation. It is therefore necessary to assess the contribution to the dose rate of each type of radiation in a particular cell. The total dose rate presented in next paragraphs is the sum of both neutron and photon contribution.

**Figure 5:**
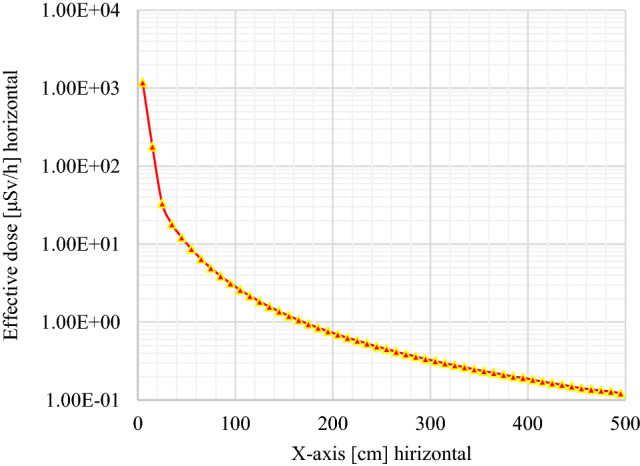
Effective dose (horizontal profile or x-axis) investigated from the tally for output results where the interval 0–23.70 cm represents the DSRSs container radius and the interval 23.70–500.00 cm corresponds to the closest area to the waste package to be disposed of.

**Figure 6:**
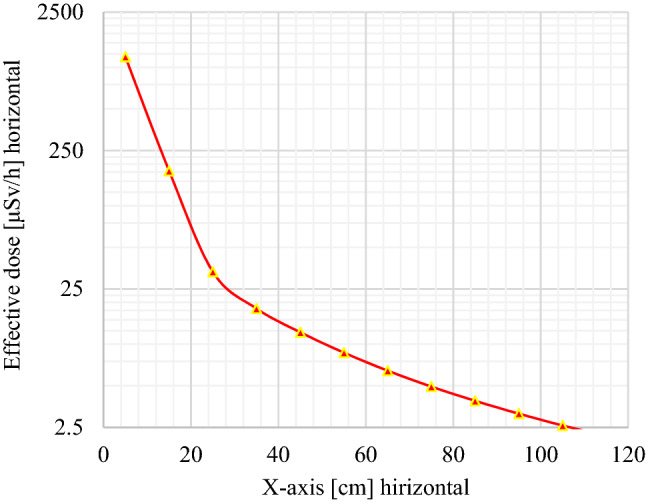
Effective dose profile inside the DSRSs container’s [from 0 to 23.70 cm] and in the closest horizontal boundary area to reach the ALARA acceptable dose limit of 2.5µSv/h for public acceptance. The slope shift observed is mainly due to the change of the medium from the waste package to the enclosure environment (filled with air at ground level).

**Figure 7:**
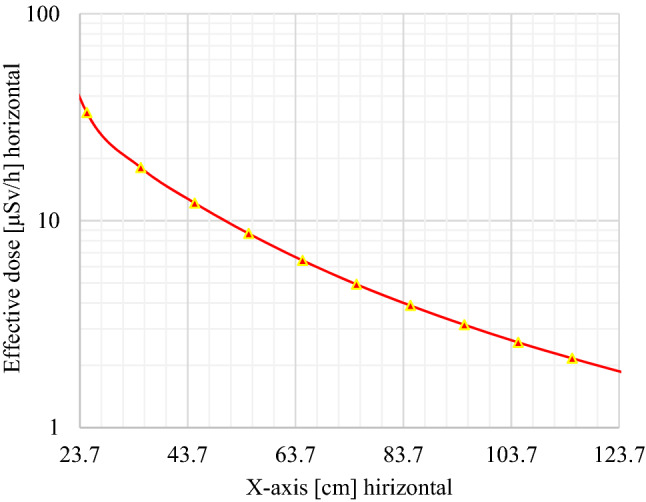
Dose evolution from the DSRSs container’s contact point to 1.00 m away. The effective dose drops from 33.310 ± 0.001 μSv/h at contact to 1.752 ± 0.004μSv/h at 1 m, which is acceptable for the ALARA hypothesis.

Monte Carlo simulations are used to optimize the DSRS disposal container in view to reduce high radiological risk to the population and the radioactive waste facility staff. To evaluate the effectiveness of shielding materials of the waste package or DSRS container used for ^241^Am/Be long-term storage, the dose profile was plotted for the decision-making process. As seen in Figs. 5, 6, and 7, the horizontal effective dose profiles around the DSRS package are displayed. From these figures, it is observed that the distance needed to reduce the effective dose rate up to the ALARA acceptable limit for the public area (2.5 μSv/h) is 84.30 cm^14,16,27,30,39,54,55^. However, it appears to be 108.00 cm on the graph displayed in Fig. 6, but the radius of the container is 23.70 cm. The result was directly associated to the tally position for the dose rate computed using the code. A volume tally was used to validate the resolution of the geometry and the obtained results were in perfect accordance with the real values with zero relative standard deviation. Therefore, it appears not necessary to perform another computation for the above-mentioned distances. These results are in accordance with the international regulations (IAEA^56–58^) for the waste drum design^59,60^. The effective dose variation displayed in Fig. 7 shows that the acceptable limit is reached in the closest of 1.00 m from the surface of the DSRS container (upper contact point). It can be concluded that the design is effective as the effective dose rate at 1 m from the contact of the DSRS container is <  ~ 2 μSv/h. Thus, the dismantling operation of DSRSs and their packaging into one Am1 P60 Capsule, sealing, and storage activities could now be implemented at the centralized disused sealed radioactive sources storage. This can now be achieved for the 8 sources presented in Table 1. The evaluation performed also shows how crucial it is to evaluate the country’s capacity in terms of Am1 P60 capsules.

**Figure 8:**
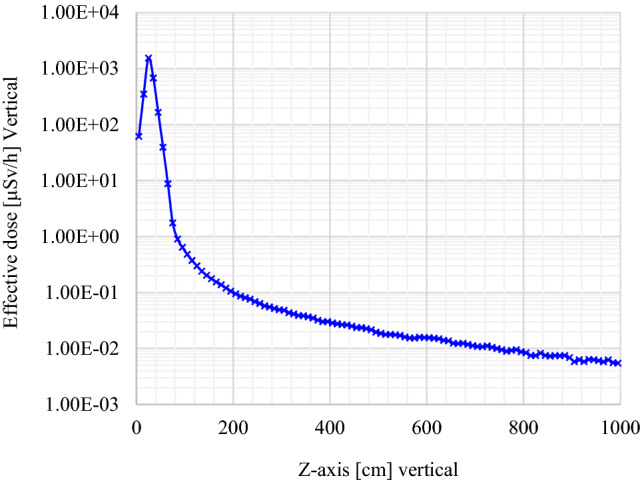
Effective dose (vertical profile or z-axis) investigated from the tally for output results where the interval 0–74.00 cm represents the DSRSs container height and the interval 74.00–1000.00 cm corresponds to the closest area on top of the encapsulated waste to be disposed of.

**Figure 9:**
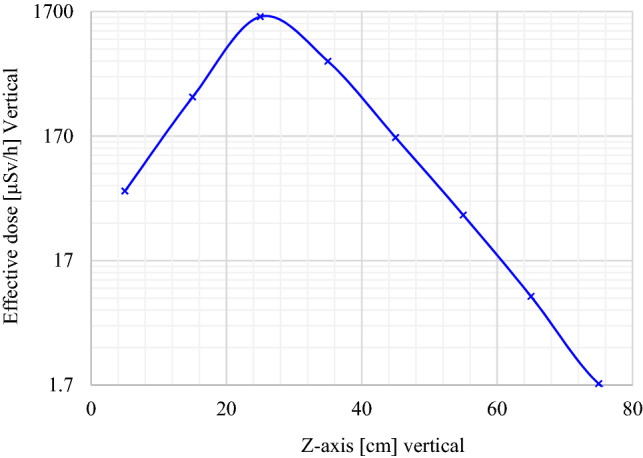
Effective dose profile inside the DSRSs container’s [from 0 to 74.00 cm] where the position of ^241^Am/Be source pre-encased in a P60 capsule encapsulated into the concrete-filled drum is set between 21.20 and 49.70 cm.

Figures 8 and 9 display the vertical effective dose profile for the decision-making process. The definition of the source between 21.20 and 49.70 cm is the main reason why the maximum (a peak) is observed in this interval. Considering the ALARA principle, the effective dose rate at the contact of the top of the DSRS container is 1.750 ± 0.004 μSv/h (seen in Fig. 8 and Table 2). This value is comparably lower than the regulatory value of 2.5 μSv/h set for public adjacent areas. The effective dose rate at 1.00 m from the top (z-axis) is observed to be 0.137 μSv/h, which is radiologically acceptable for the public.

**Table 2:** Data for decision-making process based on Monte Carlo simulation in view to achieve public exposure condition in the boundary adjacent areas to the DSRSs container. Public exposure here refers to the ALARA principle of less than 1 mSv/year, which in other words means 2.5 μSv/h.

Dose at contact (μSv/h) on x-axis horizontal	33.310 ± 0.001
Dose 1 m away (μSv/h) on x-axis horizontal	1.830 ± 0.005
Distance (cm) needed to reach 2.5 μSv/h on x-axis	84.310 ± 0.009
Dose at contact (μSv/h) on z-axis vertical	1.752 ± 0.004
Dose 1 m away (μSv/h) on z-axis vertical	0.137 ± 0.017
Distance (cm) needed to reach 2.5 μSv/h on x-axis	0.0

The obtained results also show that the optimum packaging and encapsulation of ^241^Am/Be disused sources could be preceded by the Monte Carlo assessment of the waste packages. Though the assessment for the disposal of DSRSs was done for disused ^241^Am/Be neutron sources in Cameroon, the same process could be implemented in other countries that deal with neutron sources prepared into special capsule forms. In such cases, the obtained results are useful worldwide and available for implementation by other radioactive waste management facilities. The aforementioned Monte Carlo simulation is a prior study to the dismantling operation that enhances the cradle-to-grave management of DSRSs in developing countries like Cameroon. The dismantling operation could be carried out safely at any time knowing that the effective dose rate in the enclosed areas to the DSRS container shall remain less than the recommended value of 2.5μSv/h for the public area. In addition, another work should be performed to evaluate the safety and security of the disposal site and the interim storage in the condition where many DSRS drums are stored in several stages in a confined area. It should be performed when the DSRS capability of the country in the next century is evaluated. This work also helped to evaluate the Am1 P60 capsules and the borate concrete-filled drums capacity of each developing country that has to deal with ^241^Am/Be disused sources. Such capacity definition is of primary interest in collaboration with international organizations like IAEA and bilateral cooperation for radioactive waste management (the case of US-Cameroon). From the total investigated ^241^Am/Be DSRSs, the country capacity of a Am1 P60 capsule corresponds to one borate concrete-filled drum for long-term storage purposes. A similar study is being carried on to evaluate the case of gamma emitter DSRSs as ^137^Cs, ^60^Co, available in large quantities as radioactive waste in developing countries^16,30,39^.

## Conclusions

The optimum encapsulation of ^241^Am/Be disused sealed radioactive sources based on PHITS Monte Carlo simulations for the long-term storage of DSRSs in Cameroon was performed. The shielding effectiveness of the disused source drums and Am1 P60 capsules for storage operations was evaluated by taking into consideration the total number of ^241^Am/Be DSRSs inventoried. This was to assess the minimum of radiological risk for both radioactive waste specialists (operators during the dismantling operations) and the public. The P60 special form capsules are to be produced and used in a worldwide project on the “Sustaining Cradle-to-Grave Control of Radioactive Sources”. So the characteristics of the special capsule were investigated to conclude on its properties before the future implementation of joint dismantling expert missions in participating states.

The effective radiation dose rates obtained were in agreement with the ALARA principle and regulatory requirements for the exposure rate optimization. In fact, the obtained effective dose rates were found to be 1.830 (horizontal calculation) and 0.137 μSv/h (vertical computation) which are lower than the 2.5 μSv/h acceptable limit for the public area. For the radiation dose profile of ^241^Am/Be source obtained, the neutron flux and gamma generated from neutron absorption are in accordance with the research hypothesis and fit our expectations. As the Am1 P60 capsule was designed to carry up to 10 TBq of ^241^Am/Be source, the future dismantling operations to be arranged should take into consideration all national inventoried ^241^Am/Be DSRSs. Also, means of filing the borate concrete-filled drums and the remaining space in the geometry with locally made concrete should be designed prior to the dismantling operation. As the issue of radioactive and nuclear waste management is an up-to-date topic, the future perspective will consist of looking at a Monte Carlo-based method to reduce the heat of nuclear/radioactive waste and to transmute, if possible on a small scale, long-lived radionuclides to short-lived ones. The ongoing research project is actually evaluating the Cs1 P60 capsule national capacity to dismantle gamma emitters DSRSs.

## Data Availability

The data that supports the findings of this study are available within the article and upon reasonable request.
